# Diagnostic image‐based treatment planning for online adaptive ultra‐hypofractionated prostate cancer radiotherapy with MR‐Linac

**DOI:** 10.1002/acm2.70075

**Published:** 2025-03-16

**Authors:** Yuan Xu, Ningning Lu, Qiao Li, Kuo Men, Xinming Zhao, Jianrong Dai

**Affiliations:** ^1^ Department of Radiation Oncology National Cancer Center/National Clinical Research Center for Cancer/Cancer Hospital Chinese Academy of Medical Sciences and Peking Union Medical College Beijing China; ^2^ Department of Radiology National Cancer Center/National Clinical Research Center for Cancer/Cancer Hospital Chinese Academy of Medical Sciences and Peking Union Medical College Beijing China

**Keywords:** adaptive radiotherapy, diagnostic CT, Elekta Unity, MR‐Linac

## Abstract

**Purpose:**

A new workflow was investigated for Elekta Unity MR‐Linac by removing the computed tomography (CT)‐simulation step and using diagnostic CT (DCT) for reference plan generation.

**Materials and methods:**

Ten patients with ultra‐hypofractionated prostate cancer treated with magnetic resonance imaging (MRI)‐guided adaptive radiotherapy were retrospectively enrolled. Targets and organs at risk (OARs) were recontoured on DCT, and Hounsfield unit conversions to relative electron density were calibrated for DCT. Reference plans were reoptimized and recalculated using DCT for Unity. Subsequent adaptive plans were designed through an adapt‐to‐shape workflow to edit targets and OARs via daily MRI to generate a new treatment plan. Bulk electron density information for Unity adaptive plan was compared between planning CT (PCT) and DCT for volumes of interest. Dosimetric parameters were evaluated between PCT‐ and DCT‐based reference and adaptive plans for target coverage and OAR dose constraints.

**Results:**

Bulk relative electron density differences between PCT and DCT were within ±1% for targets and OARs, excepting the rectum. PCT and DCT‐based reference plans did not significantly differ in mean target coverages or for OARs in dosimetric difference except for *V*_36 Gy_ of the rectum. PCT‐ and DCT‐based adaptive plans did not significantly differ for most dosimetric parameters of targets and OARs except for *V*_29 Gy_ and *V*_36 Gy_ of the rectum, *V*_18.1 Gy_ of the bladder, and *D*_50%_ of the urethra.

**Conclusions:**

By removing the CT simulation step, it is feasible to use DCT for designing reference and adaptive plans in the Unity ATS workflow. The workflow increased adaptive radiotherapy efficiency and decreased patient waiting time and additional radiation dose.

## 1 INTRODUCTION

Radiotherapy is a common treatment modality for prostate cancer. Ultra‐hypofractionated (four to seven, but commonly, and herein, five fractions) radiotherapy can reduce the treatment period to less than 2 weeks compared to the 2 months conventionally fractionated schedule.^1^, ^2^ However, the ultra‐hypofractionated approach for prostate cancer enhances concerns for toxicity to organs‐at‐risk (OARs) such as the bladder and rectum due to intra‐fraction organ mobility.^3^ Additionally, there is an increased concern for ensuring target coverage for the same reason as follows: that a geometric miss using the shorter treatment schedule is less likely to be resolved by the averaging of the dose distribution on subsequent fractions.^4^ Both online adaptive magnetic resonance imaging‐guided radiotherapy (MRIgRT) or the use of fiducial markers were reported to improve the quality of care for many treatments by ensuring consistent geometric accuracy compared to non‐adaptive cone beam computed tomography (CBCT)‐guided treatments without fiducial markers^5^, ^6^; the MRIgRT provides enhanced soft tissue contrast and no extra radiation exposure compared to CBCT.^7^

While adaptive MRIgRT technology brings advantages of monitoring targets and OARs with high contrast in real time,^8^ the treatment planning and delivery workflows remain complex, resource intensive, and would benefit from improvement. In short, the MRIgRT adaptive workflow differs from conventional radiotherapy workflows because the treatment plan, that is, generated during the planning process is used as a reference plan, but may never actually be delivered because the treatment is adapted each fraction, and new plans are generated and delivered based on the daily anatomy.^9^

CT simulation is typically necessary for external beam radiotherapy treatments partly because: (1) the CT simulator couch more closely mimics the treatment couch, (2) the patient is set up in the treatment position and immobilized, (3) the CT sim scan protocol is characterized so that the HU response is plotted versus the electron or physical density.^10^ This allows the treatment planning system to calculate the absorbed dose. The adaptive radiotherapy adapt‐to‐shape (ATS) workflow, that is, available on the Elekta unity MR‐Linac can account for the first two issues, which partially nullifies the need for CT simulation.

In the context of conventional x‐ray‐guided radiotherapy, diagnostic CT image‐based treatment planning (without CT sim) has been reported for palliative and emergent radiotherapy (sometimes with heterogeneity corrections turned off for simplicity).^11^, ^12^ Such a change to the process can result in significantly shorter waiting times between when the patient is identified for radiotherapy and the treatment is delivered. In one case, it was shown that the patient could be treated in the palliative setting in only 28 min by removing the CT simulation step.^11^ Despite difference between CT simulation and diagnostic CT scans that typically require the CT simulation step, it is worth investigating the technical and dosimetric feasibility of removing the CT simulation step for online adaptive MRIgRT, hoping that the total length of the MRIgRT workflow could be reduced. The current study investigates the dosimetric feasibility of improving workflow efficiency by removing the CT simulation step of the treatment planning process and use CT data from routine diagnostic CT scans for the reference plan.

## 2 MATERIALS AND METHODS

### 2.1 Patient information

Ten patients with intermediate to high‐risk localized prostate cancer who had been treated with Unity MR‐Linac were enrolled. This retrospective study was approved by the ethical review board and informed consent was waived. The mean age of the 10 male patients was 73.6 years (range 52–88 year), and their average weight was 70.4 kg (range 63–83 kg).

### 2.2 CT simulator and diagnostic CT

**FIGURE 1:**
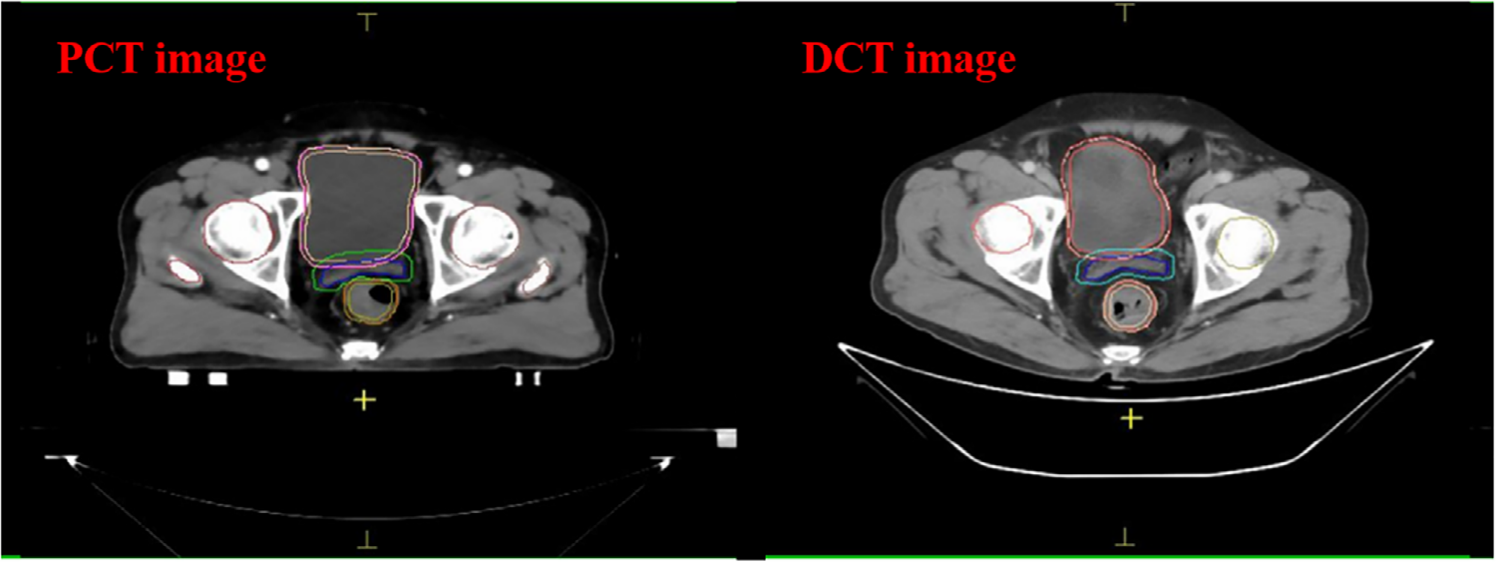
Illustration of PCT image (left) and DCT image (right).

All patients received both DCT scanning before radiotherapy workflow. The DCT of patients were scanned using diagnostic CT from various vendors (GE, Philips, Siemens). It is shown in Figure 1 that DCT has a curved couch comparing with a flat couch of CT simulator. Moreover, patients were generally not immobilized during DCT scanning, and the HU to relative electron density conversion was not calibrated for DCT.

**FIGURE 2:**
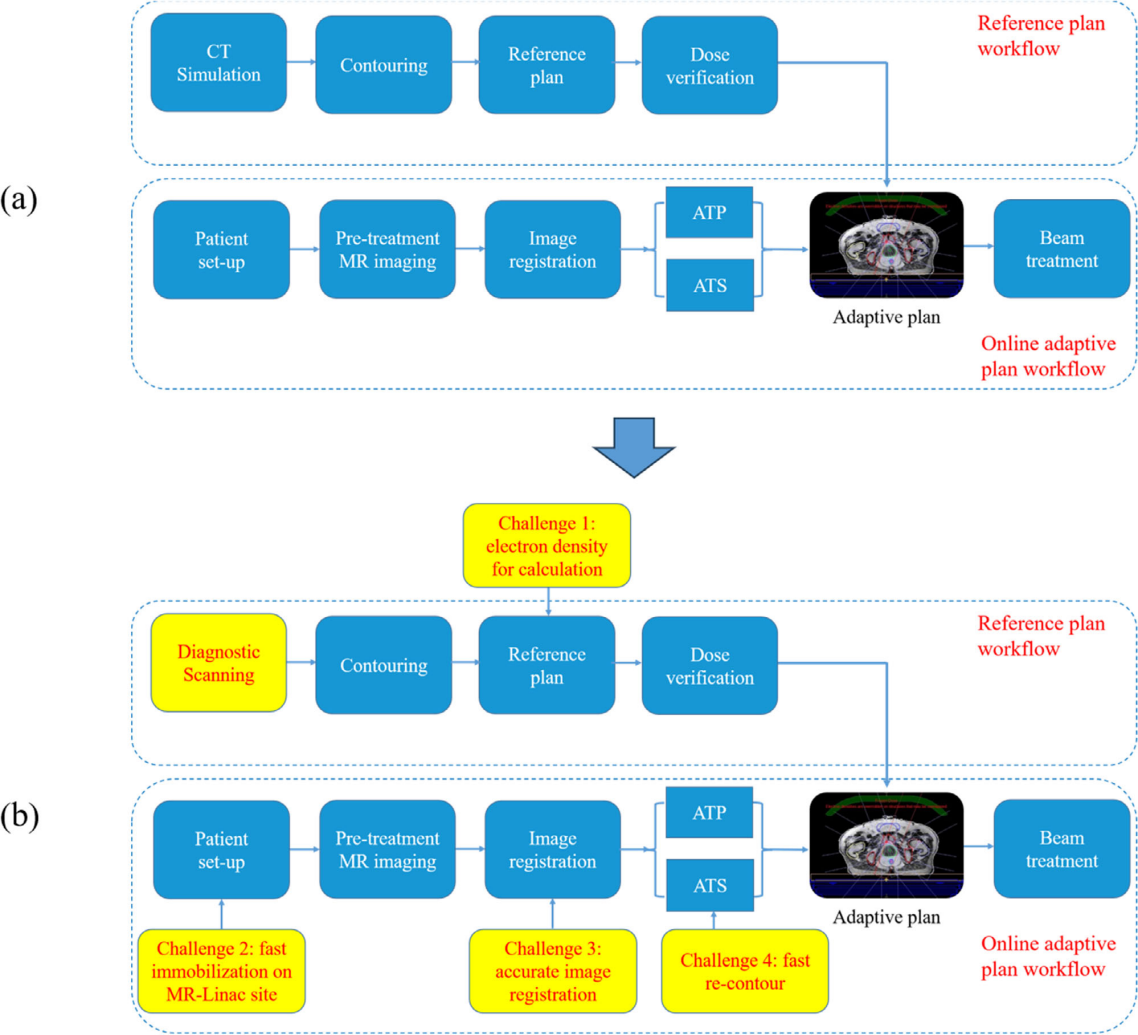
The PCT‐based (a) and DCT‐based (b) workflow of Unity reference plan and adaptive plan designing.

**FIGURE 3:**
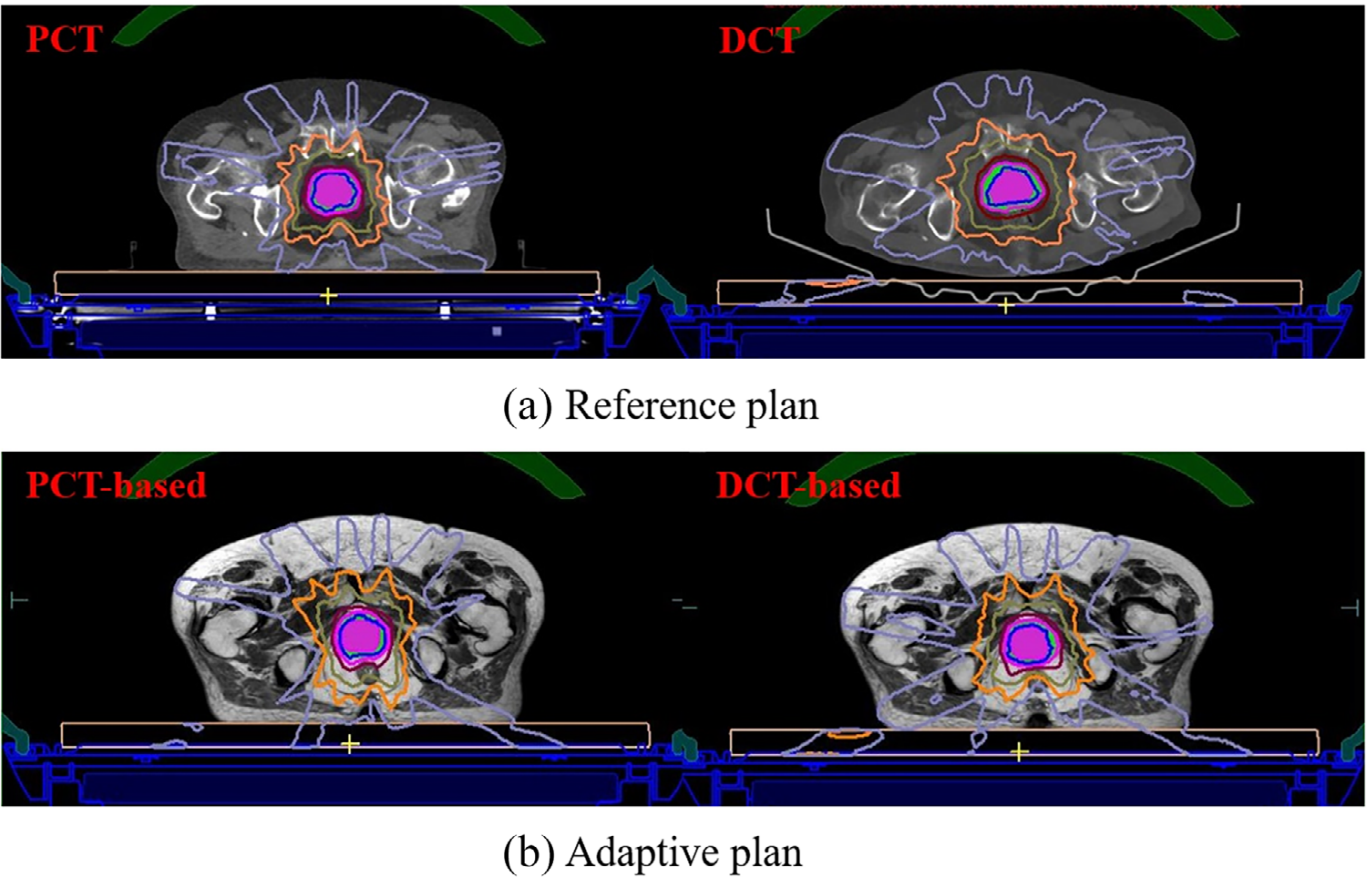
Dose distribution of PCT and DCT‐based reference plan (a) and adaptive plan (b).

### 2.3 The Elekta Unity MRIgRT platform

The treatment platform used in this investigation is the Elekta Unity MR‐Linac (Elekta, Crawley, UK),^13^ an MRIgRT platform that is used for online adaptive radiotherapy workflows. The treatment unit is a 7‐MV flattening‐filter free linac, that is, coupled to a 1.5 T MR image guidance system, that is, used for pretreatment imaging and intra‐fraction motion management.^13^ Unity is capable of delivering fixed‐gantry IMRT fields with step‐and‐shoot MLCs, but no VMAT treatments. Initial treatment planning is performed offline in the Monaco treatment planning system (Elekta AB, Stockholm, Sweden), and the online adaptive planning is performed in an overhauled version of Monaco, that is, developed specifically for online adaptations with the Unity.^9^ There are unique aspects of contouring and treatment planning that will be discussed in subsequent sections.

The platform allows for two types of online adaptive workflows: ATP and ATS. Briefly, the ATP workflow is “simpler,” and generally used for prostate adaptations when using a CT simulation‐based workflow because usually the beam segment weights and shapes simply need to be morphed and adjusted to the target or patient at the new position (both relative to the isocenter). The ATS workflow is more appropriate for this study (at least for the first fraction) since the patient's anatomy likely will be changed between the reference dataset and the daily image due to immobilization and a flat couch top compared to the DCT. In such a case while using ATS, the contours may be adapted from a reference plan to the daily anatomy, and adjusted or recontoured, through a registration between the reference plan image set and daily MRI. ATP does not allow the contours to be adjusted during the adaptation. The ATS and ATP workflows are described further in prior works.^9^

### 2.4 Contouring

DCT images were preprocessed with MIM software (version 7.1.4, MIM Software, OH, USA) to compatible with TPS, such as medical record number, patient position or image resolution, then transferred to the Monaco TPS. Targets and OARs were recontoured on DCT using a fusion of the diagnosic MR image to aid contouring. The same radiation oncologist who treated the patient performed this retrospective contouring. The clinical target volume (CTV_3625) was defined as the whole prostate (Prostate GTV) with a margin of 3 mm (0 mm posteriorly) for extraprostatic extension.^14^ The proximal 1 cm SVs were included in CTV_3625 for patients with high SV involvement rate, and for patients with minimal T3b, the whole SV was included according to the National comprehensive.^14^ The planning target volume (PTV_3625) was generated by expanding a uniform 3‐mm margin to CTV_3625. PTV_4000 was defined as prostate with a 1‐mm contraction for simultaneous boost. The boost volume is a patient‐specific focal boost, which is performed on occasion.^15^ Contouring was also performed on the adjacent OARs such as the rectum, rectum wall, bladder, bladder wall, bone, and femur heads. As the couch of the DCT was curved and not planar, it was contoured and defined with a forced relative electron density of 0.01. The couch was then replaced with a mattress and treatment couch of Unity in the TPS.

### 2.5 Electron density

The reference CT provides the electron density information, which is needed to calculate dose, and to generate the reference plan and is retained for the adaptations. Therefore, the HU to ED characterization is necessary. Since this study evaluates the use of DCT, HU to relative electron density for the eight DCT scanners was characterized using a CIRS 062 M  electron density phantom (CIRS Inc., Norfolk VA, USA) (PCT is already characterized). The phantom, which contains plugs with known relative electron densities, was scanned in each scanner, and a plot of relative electron density versus HU was made for the planning system to compute the dose from the HU. For different DCT scanning, a separate relative electron density was utilized for dose calculation.

During the adaptive planning, each structure is assigned a relative electron density (relative to water) and a priority relative to the other structures. The relative electron density for the structure is computed automatically using the average ED value of the structure on the reference CT data. The priority of the structure is user‐defined and is needed to handle dose calculation for structures that overlap but have different average electron densities. The prioritization of the structures in the TPS is known as “layers.” These ED assignments and layers that are determined during reference planning are retained in the structure set and planning parameters that are imported to the online system during the adaptive phase, since the adaptive phase only has MR image guidance and therefore needs density assignments to calculate dose. The differences of bulk electron densities between the PCT and the DCT were evaluated.

### 2.6 Treatment planning

The prescription dose is 36.25 Gy in five fractions to PTV_3625 with a simultaneous integrated 40 Gy boost to PTV_4000. The dosimetric constraints for OARs are shown in Table 1.^14^, ^16^, ^17^ Reference treatment plans were developed using the PCT‐based conventional workflow shown in Figure 2a. DCT plans were generated by substituting the DCT data in place of the PCT data in otherwise the same workflow (Figure 2b).

**TABLE 1:** Dosimetric constraints of ultra‐hypofractionated radiotherapy for prostate cancer patients.

Volume of interest	Parameters	Requirements	Volume of interest	Parameters	Requirements
PTV_4000	*V*_40 Gy_ (%)	90%–95%	Rectum	*V*_18.1 Gy_ (%)	<40%
PTV_3625	*V*_36.25 Gy_ (%)	90%–95%		*V*_29 Gy_ (%)	<20%
	*V*_34.4 Gy_ (%)	>98%		*V*_36 Gy_ (cm^3^)	<1–2 cm^3^
	*V*_48 Gy_ (cm^3^)	<1–2 cm^3^	Femur head L	*V*_14.5 Gy_ (%)	<5%
Rectum wall	*D*_max_ (Gy)	<40 Gy	Femur head R	*V*_14.5 Gy_ (%)	<5%
	*V*_18.1 Gy_ (%)	<40%	Bladder wall	*V*_18.1 Gy_ (%)	<50%
	*V*_29 Gy_ (%)	<20%		*V*_37 Gy_ (cm^3^)	<10–20 cm^3^
	*V*_36 Gy_ (cm^3^)	<1–2 cm^3^	Bladder	*V*_18.1 Gy_ (%)	<50%
	*V*_38 Gy_ (cm^3^)	<0.1–0.2 cm^3^		*V*_37 Gy_ (cm^3^)	<10–20 cm^3^
Urethra	*D*_50%_ (Gy)	<42–45 Gy			

The intensity‐modulated radiotherapy (IMRT) plans were generated with online version of Monaco TPS (version 5.4, Elekta, Crawley, UK), which supported online adaptive plan designing for Unity. Generally, 9–15 fields were used according to the complexity of planning.

Then, adaptive plans were generated with the pre‐treatment MR scanned before, the contours and images were identical to PCT‐based adaptive plans, and electron density information and initial optimization parameters were from DCT‐based reference plans.

In this study, ATS adaptive workflow was adopted during all treatment fractions because of concerns of obvious interfractional variation of contours. The five fractions of ATS adaptive plans per patient were compared between initial DCT‐based reference plan and the PCT‐based adaptive plans.

The potential challenges were also illustrated in Figure 2b, including electron density for calculation, fast immobilization on MR‐linac site, accurate image registration, and fast re‐contour, etc. In this study, the dosimetric parameters were investigated as the first step.

### 2.7 Analysis and statistical methods

The dosimetric metrics in Table 1 and monitor unit (MU) were compared for DCT‐based and PCT‐based reference plans. The adaptive plans generated with DCT and PCT were also compared with dosimetric parameters and MU.

Statistical analyses of bulk relative electron density and dosimetric parameters between PCT‐ and DCT‐based plans were performed using SPSS (version 19.0, IBM, New York, NY, USA) to demonstrate the statistical significance. For data with a normal distribution, a paired sample *t*‐test was applied to test significance; otherwise, the nonparametric Wilcoxon signed‐rank test was used. Differences were considered significant with a *p*‐value of <0.05.

## 3 RESULTS

### 3.1 Electron density

For calibrating CT HU to relative electron density, the differences between PCT and DCT were relatively small for the lower electron density zone (approximately 0–1.1) and increased with increasing relative electron density. The bulk relative electron density from both PCT and DCT is shown in Table 2. For volumes, the relative difference between PCT and DCT was within ±1% except for the rectum, and the differences were not significant except for femur heads.

**TABLE 2:** Comparison of bulk relative electron density (mean ± standard deviation) from PCT and DCT.

Volume of interest	Relative electron density from PCT	Relative electron density from DCT	Relative difference (%)	*p*
Urethra	1.039 ± 0.003	1.046 ± 0.001	0.65	0.168
PTV_4000	1.036 ± 0.004	1.039 ± 0.011	0.34	0.539
PTV_3625	1.029 ± 0.005	1.034 ± 0.013	0.51	0.539
Rectum wall	1.002 ± 0.014	1.009 ± 0.018	0.64	0.344
Rectum	0.979 ± 0.050	1.003 ± 0.025	2.5	0.103
Femur head L	1.124 ± 0.021	1.134 ± 0.028	0.93	0.002
Femur head R	1.122 ± 0.020	1.131 ± 0.025	0.85	0.010
Bladder wall	0.998 ± 0.007	1.005 ± 0.018	0.75	0.232
Bladder	1.008 ± 0.006	1.010 ± 0.014	0.16	0.920
Patient	0.980 ± 0.009	0.973 ± 0.035	0.69	0.826

### 3.2 Comparison of reference plans based on PCT and DCT

The reference plan dose distribution of one patient based on the PCT and DCT is shown in Figure 3a. The dosimetric parameters of targets and OARs and MU were compared in Table 3. For these indexes, significant differences were absent between PCT‐ and DCT‐based reference plans excepting that differences were significant for the absolute volume of 36 Gy in the rectum (*p *< 0.05), although the difference was relatively small and within the dose constraint for the clinic.

**TABLE 3:** Comparison of dosimetric parameters (mean ± standard deviation) between PCT and DCT‐based reference plans.

Volume of interest	Parameters	PCT‐based plan	DCT‐based plan	*p*	Pass or fail constraint
PTV_4000	*V*_40 Gy_ (%)	93.0% ± 2.3%	93.8% ± 1.9%	0.441	Pass
PTV_3625	*V*_36.25 Gy_ (%)	94.7% ± 1.9%	95.4% ± 1.4%	0.285	Pass
	*V*_34.4 Gy_ (%)	99.1% ± 0.9%	99.2% ± 0.5%	0.441	Pass
	*V*_48 Gy_ (cm^3^)	0 cm^3^	0 cm^3^	–	Pass
Rectum wall	*D*_max_ (Gy)	38.3 ± 0.7 Gy	38.7 ± 0.7 Gy	0.203	Pass
	*V*_18.1 G_y (%)	32.0% ± 7.0%	32.5% ± 6.3%	0.721	Pass
	*V*_29_ _Gy_ (%)	15.7% ± 5.8%	17.1% ± 4.6%	0.445	Pass
	*V*_36 Gy_ (cm^3^)	0.79 ± 0.47 cm^3^	0.99 ± 0.66 cm^3^	0.386	Pass
	*V*_38 Gy_ (cm^3^)	0.038 ± 0.038 cm^3^	0.066 ± 0.075 cm^3^	0.161	Pass
Rectum	*V*_18.1 Gy_ (%)	29.7% ± 8.9%	29.5% ± 7.5%	0.508	Pass
	*V*_29 Gy_ (%)	11.4% ± 5.3%	12.0% ± 4.4%	0.799	Pass
	*V*_36 Gy_ (cm^3^)	0.61 ± 0.39cc	1.01 ± 0.66 cm^3^	0.047	Pass
Femur head L	*V*_14.5 Gy_ (%)	1.18% ± 1.42%	2.16% ± 1.44%	0.086	Pass
Femur head R	*V*_14.5 Gy_ (%)	1.18% ± 1.36%	1.87% ± 1.56%	0.263	Pass
Bladder wall	*V*_18.1 Gy_ (%)	22.4% ± 10.8%	25.4% ± 7.4%	0.241	Pass
	*V*_37 Gy_ (cm^3^)	3.0 ± 1.3 cm^3^	2.9 ± 1.1 cm^3^	0.721	Pass
Bladder	*V*_18.1 Gy_ (%)	19.0% ± 11.8%	25.1% ± 7.6%	0.203	Pass
	*V*_37 Gy_ (cm^3^)	6.0 ± 3.0 cm^3^	8.0 ± 3.5 cm^3^	0.059	Pass
Urethra	*D*_50%_ (Gy)	41.0 ± 0.7 Gy	41.0 ± 0.4 Gy	0.646	Pass
	Monitor unit	2418 ± 704 MU	2484 ± 462 MU	0.765	–

### 3.3 Comparisons of ATS adaptive plans between PCT and DCT

The dose distribution of one ATS plan optimized with PCT‐ and DCT‐based reference plans is shown in Figure 3b. The dose distributions are similar between PCT‐ and DCT‐based adaptive plans. The dosimetric parameters and MU of adaptive plans are shown in Table 4, and significant differences were absent for dosimetric parameters of targets and OARs except for *V*_29 Gy_ (%) and *V*_36 Gy_ (cm^3^) of the rectum, *V*_18.1 Gy_ (%) of the bladder, and *D*_50%_ of the urethra. The differences for these parameters were also small and within the dose constraint for the clinic.

**TABLE 4:** Comparison of dosimetric parameters (mean ± standard deviation) between PCT and DCT‐based adaptive plans.

Volume of interest	Parameters	PCT‐based plan	DCT‐based plan	*p*	Pass or fail constraint
PTV_4000	*V*_40 Gy_ (%)	94.5% ± 2.4%	94.0% ± 1.9%	0.067	Pass
PTV_3625	*V*_36.25 Gy_ (%)	95.3% ± 2.5%	96.5% ± 1.9%	0.086	Pass
	*V*_34.4 Gy_ (%)	99.3% ± 0.6%	99.6% ± 0.5%	0.120	Pass
	*V*_48 Gy_ (cm^3^)	0 cm^3^	0 cm^3^	–	Pass
Rectum wall	*D*_max_ (Gy)	38.2 ± 1.0 Gy	37.4 ± 5.4 Gy	0.328	Pass
	*V*_18.1 Gy_ (%)	30.4% ± 7.4%	29.4% ± 7.0%	0.182	Pass
	*V*_29 Gy_ (%)	14.9% ± 5.3%	14.8% ± 5.0%	0.710	Pass
	*V*_36 Gy_ (cm^3^)	0.54 ± 0.38 cm^3^	0.64 ± 0.36 cm^3^	0.013	Pass
	*V*_38_ _Gy_ (cm^3^)	0.029 ± 0.064 cm^3^	0.019 ± 0.032 cm^3^	0.161	Pass
Rectum	*V*_18.1 Gy_ (%)	28.6% ± 8.1%	28.2% ± 8.5%	0.544	Pass
	*V*_29 Gy_ (%)	10.8% ± 4.8%	11.3% ± 4.6%	0.08	Pass
	*V*_36 Gy_ (cm^3^)	0.41 ± 0.30 cm^3^	0.49 ± 0.29 cm^3^	0.018	Pass
Femur head L	*V*_14.5 Gy_ (%)	1.53% ± 1.69%	1.85% ± 1.69%	0.29	Pass
Femur head R	*V*_14.5 Gy_ (%)	1.18% ± 1.45%	1.51% ± 1.50%	0.204	Pass
Bladder wall	*V*_18.1 Gy_ (%)	16.1% ± 7.2%	16.3% ± 7.6%	0.589	Pass
	*V*_37 Gy_ (cm^3^)	2.21 ± 1.16 cm^3^	2.22 ± 1.15 cm^3^	0.933	Pass
Bladder	*V*_18.1 Gy_ (%)	14.5% ± 7.9%	15.2% ± 8.4%	0.067	Pass
	*V*_37 Gy_ (cm^3^)	5.1 ± 3.6 cm^3^	5.2 ± 3.4 cm^3^	0.753	Pass
Urethra	*D*_50%_ (Gy)	40.9 ± 0.9 Gy	40.6 ± 0.6 Gy	0.001	Pass
	Monitor unit	2463 ± 407 MU	2475 ± 408 MU	0.886	

## 4 DISCUSSION

Herein, the dosimetric feasibility of a new workflow was investigated by removing the CT simulation step, therefore, potentially increasing the workflow efficiency and reducing the radiation used for CT scanning. MRIgRT can benefit online adaptive radiotherapy and allows consecutive monitoring of anatomical variations and adaption of the treatment plan to daily MRI, which reflects updated contours just before treatment. This technique is especially suitable for abdominal tumors that have obvious organ motions and need improved soft tissue contrast.^18^ However, the workflow is complex and time‐consuming. Herein, the feasibility of generating the reference and adaptive plans based on DCT was demonstrated, and the influence of HU on relative electron density conversion was found to be non‐negligible between the CT simulator and diagnostic CT.

The ATS adaptive workflow nullifies the reference fluence and segments and reoptimizes both parameters on each adaptive fraction based on the daily treatment anatomy. In contrast to the ATP workflow, this feature allows ATS to handle changes to the shapes of the structures, including differences that may be present from using different (or nonexistent) immobilization and a different couch top on the reference dataset compared to the treatment dataset,^19^ which had been two of the tenets necessitating a CT simulator. As daily MRI through Unity ATS workflow allows editing of contours, and the contours on the CT image of the reference plan are not the final contours for planning. Thus, even though the position and anatomical structures of patients differ between PCT and DCT scanning, DCT still has potential use in reference plan designing. However, after removing the CT simulation step, the positioning and immobilization of patients during treatment must still be considered. For diagnostic scanning, patients are normally not immobilized. During adaptive treatment, patients must be immobilized to avoid intrafraction motion, which can be realized by using an MRI‐compatible vacuum bag or foam cushion.^20^, ^21^ Furthermore, the time required for contouring of the adaptive plan may increase comparing DCT‐ to PCT‐based plans due to potential larger anatomical differences (e.g., body recontouring) between DCT and pre‐treatment MR. The treatment couch should also be relocated as the position of the couch was unknown with DCT scanning, and the position could be corrected with daily MR scanning.

**FIGURE 4:**
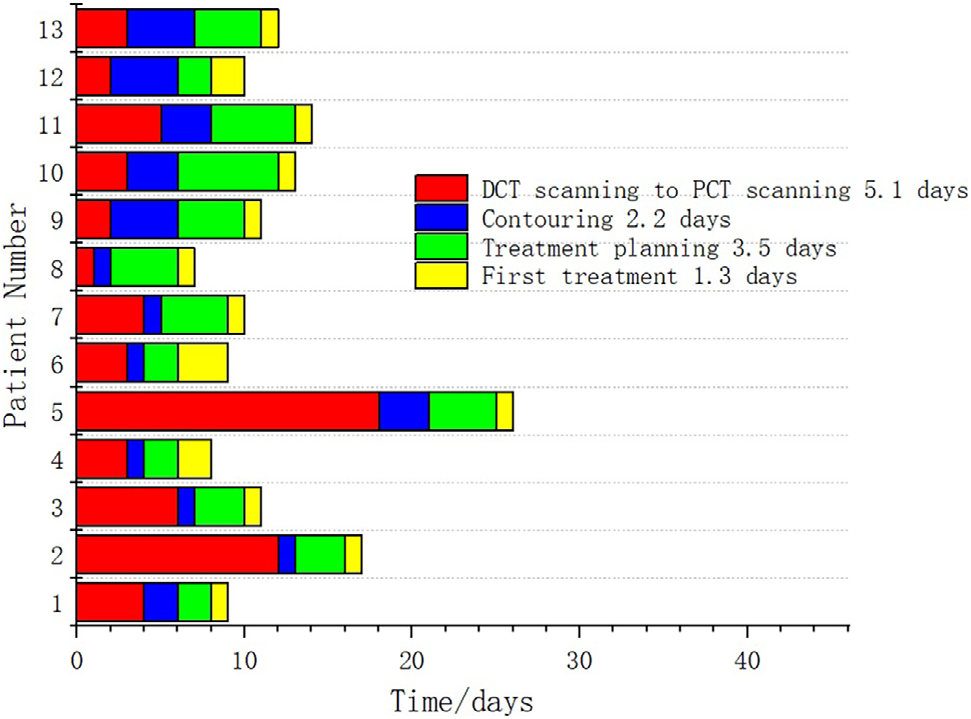
The time for different phases of radiotherapy for prostate cancer patients treated with MR‐Linac.

As shown in Figure 4, the treatment period for prostate cancer patients using Unity from scanning of DCT to beginning of radiotherapy were evaluated, and it was approximately average 5.4 working days (range 1–18 working days) from scanning of DCT to scanning of PCT in our institute. This period can be several weeks or even months in some busy institutes. Therefore, contouring of targets and OARs and planning of treatment can be initiated after scanning DCT to reduce the waiting time for patients. Meanwhile, the extra radiation dose of CT scanning can subsequently be reduced by removing the CT simulation step. This workflow maybe also interesting to CT‐guided adaptive radiotherapy to increase the efficiency.^22^

For MR‐Linac Unity, Carr et al. utilized prostate‐specific membrane antigen positron emission tomography/CT (PSMA‐PET/CT) scans and population‐based relative electron density for planning.^23^ However, PET/CT is not available for all patients. Therefore, a more general workflow with conventional diagnostic scans needs to be investigated. For various CTs, the conversion of HU to relative electron density can differ even though the CTs were manufactured by the same vendor. Furthermore, the structure and scanning parameters could also differ and can affect the electron density. Herein, conversion curves were measured for eight DCT from our diagnostic department. The differences between PCT and DCT from same vendor were relatively small in a low‐density zone (about 0–1.1) and increased with increasing relative electron density. For CT from different vendors, the differences could be larger. After calibrating the conversion curve, the bulk electron density of volumes between the PCT‐ and DCT‐based images was within ±1% except for the rectum. For the rectum, the difference of relative electron density was mainly caused by the volume of the air cavity and of any water present and the motion of the rectum. Nakao et al. reported a tolerance of 2% for relative electron density conversion.^24^ If no high‐density tissue (>1.1) is close to the targets, the electron density conversion curve of planning CT may be used for planning directly if the planning and DCT were made from the same vendor and used similar scanning parameters. However, for prostate cancer, the bone structure, which can have a relatively high electron density, is close to the targets, and it is better to measure the conversion curve for DCT specifically for planning.

For reference plans, the dose to OARs was not significantly different between the PCT‐ and DCT‐based plans except for *V*_36 Gy_ of the rectum. However, for reference plans, there was a bias towards high doses with DCT‐based plans shown in Table 3. This may because that the patients were normally asked to drink 300–500 mL water to ensure the filling of the bladder, which could spare bladder and rectum. But for DCT scanning, patients were generally not asked to drink water, which made it hard to reach the dosimetric constraints for the rectum and bladder. Therefore, in some cases, the physicist needed to loosen the constraints for the bladder, rectum, and other OARs. But for adaptive plans, the contours were identical as the same daily MR was used for adaptive planning, and the dose differences were minimum due to differences in initial optimization parameters and electron density.

The reference plan only provided initial optimization parameters for adaptive plans, and the dose difference between PCT‐ and DCT‐based reference plans would not affect the final dose to be delivered directly. Dosimetric parameters were similar for contours for 50 adaptive ATS plans when comparing DCT‐ and PCT‐based plans. Although the ATS workflow was implemented for patients with prostate cancer in this study according to our institution protocols,^14^ the ATP workflow was also possible after the first adaptive fraction following the general strategy selection principle to determine whether to adopt ATS or ATS workflow for adaptive radiotherapy.^25^ This study has several limitations. The number of patients was limited as MRI‐guided radiotherapy is a new developed technology and time‐consuming. The proposed workflow is also unsuitable for patients who have no DCT scanning before simulation CT scanning.

## 5 CONCLUSIONS

For targets and OARs, the difference of bulk electron density from PCT and DCT was within ±1%, excepting the rectum. For dosimetric parameters, the PCT and DCT‐based reference plans and adaptive plans did not significantly differ, excepting *V*_36 Gy_ of the rectum for reference plans and *V*_29 Gy_ and *V*_36 Gy_ of the rectum, *V*_18.1 Gy_ of the bladder, and *D*_50%_ of the urethra for adaptive plans. All dosimetric parameters were acceptable for clinical treatment. Therefore, by removing the CT simulation step, it is feasible to use DCT for designing reference and adaptive plans in the Unity ATS workflow. This approach is more efficient and reduces the extra radiation dose for patients and the workload for the CT simulation step.

## AUTHOR CONTRIBUTIONS

**Yuan Xu and Ningning Lu**: Conceptualization; methodology; writing—original draft preparation. **Qiao Li and Kuo Men**: Resources; data curation; writing—review and editing. **Jianrong Dai and Xinming Zhao**: Supervision; funding acquisition; writing—review and editing.

## CONFLICT OF INTEREST STATEMENT

The authors declare no conflicts of interest.

## ETHICAL APPROVAL AND CONSENT TO PARTICIPATE

This retrospective study was approved by the review board of Cancer Hospital, Chinese Academy of Medical Sciences, and informed consent was waived.
